# *Plasmodium falciparum* Adhesins Play an Essential Role in Signalling and Activation of Invasion into Human Erythrocytes

**DOI:** 10.1371/journal.ppat.1005343

**Published:** 2015-12-22

**Authors:** Wai-Hong Tham, Nicholas T. Y. Lim, Greta E. Weiss, Sash Lopaticki, Brendan R. E. Ansell, Megan Bird, Isabelle Lucet, Dominique Dorin-Semblat, Christian Doerig, Paul R. Gilson, Brendan S. Crabb, Alan F. Cowman

**Affiliations:** 1 The Walter & Eliza Hall Institute of Medical Research, Parkville, Victoria, Australia; 2 Department of Medical Biology, The University of Melbourne, Parkville, Victoria, Australia; 3 Burnet Institute, Melbourne, Victoria, Australia; 4 Department of Microbiology, Monash University, Melbourne, Victoria, Australia; 5 Department of Biochemistry and Molecular Biology, School of Biomedical Sciences, Monash University, Clayton, Victoria, Australia; 6 INSERM UMR_S 1134, Université Paris Diderot, GIP INTS, Paris, France; 7 Department of Immunology, Monash University, Melbourne, Victoria, Australia; 8 Department of Microbiology & Immunology, The University of Melbourne, Parkville, Victoria, Australia; MRC National Institute for Medical Research, UNITED KINGDOM

## Abstract

The most severe form of malaria in humans is caused by the protozoan parasite *Plasmodium falciparum*. The invasive form of malaria parasites is termed a merozoite and it employs an array of parasite proteins that bind to the host cell to mediate invasion. In *Plasmodium falciparum*, the erythrocyte binding-like (EBL) and reticulocyte binding-like (Rh) protein families are responsible for binding to specific erythrocyte receptors for invasion and mediating signalling events that initiate active entry of the malaria parasite. Here we have addressed the role of the cytoplasmic tails of these proteins in activating merozoite invasion after receptor engagement. We show that the cytoplasmic domains of these type 1 membrane proteins are phosphorylated *in vitro*. Depletion of PfCK2, a kinase implicated to phosphorylate these cytoplasmic tails, blocks *P*. *falciparum* invasion of red blood cells. We identify the crucial residues within the PfRh4 cytoplasmic domain that are required for successful parasite invasion. Live cell imaging of merozoites from these transgenic mutants show they attach but do not penetrate erythrocytes implying the PfRh4 cytoplasmic tail conveys signals important for the successful completion of the invasion process.

## Author Summary

Malaria parasites must invade red blood cells to survive within the human host. Members of the erythrocyte binding-like (EBL) and reticulocyte binding-like (Rh) protein families, which are present at the apical tip of merozoites as single-pass transmembrane proteins, mediate recognition of red blood cells. Although extracellular domains of *Plasmodium* adhesins are required for binding red blood receptors, only the cytoplasmic region is in contact with the parasite’s cellular machinery to initiate invasion. Therefore any signal that is initiated upon binding must be communicated via the cytoplasmic domain to other targets within the malaria parasites. We investigate the role of adhesin phosphorylation in the *P*. *falciparum* invasion process. We show that the majority of adhesin cytoplasmic tails are phosphorylated *in vitro*. Mutational analyses provide identification of the important residues required for phosphorylation and suggest that PfCK2 may be responsible. Using a conditional knockdown line, we show that PfCK2 is essential for parasite growth and invasion. Using transgenic lines, we show that mutation of the PfRh4 cytoplasmic tail results in defects in parasite invasion. This work describes the critical residues important for signaling events and implicates PfCK2 as the essential kinase involved in *P*. *falciparum* invasion.

## Introduction

The most lethal form of malaria in humans is caused by *Plasmodium falciparum*. The merozoite form of this parasite invades red blood cells beginning with initial recognition and attachment to erythrocytes (reviewed in [1]. This interaction is dynamic and involves considerable deformation of the erythrocyte membrane as the parasite rolls across the host cell surface [2]. After initial attachment, the merozoite reorientates such that its apical prominence is closely juxtaposed with the erythrocyte surface. This allows erythrocyte-binding ligands (also known as adhesins) of the parasite to interact with specific erythrocyte receptors mediating irreversible attachment and commitment to invasion. Following engagement of adhesins to their host receptors, a tight junction is formed between parasite and the erythrocyte membrane. Active invasion proceeds through an invagination of the erythrocyte surface and the tight junction moves from the apical to posterior pole of the merozoite, powered by the parasite’s actomyosin motor (reviewed in [3]). Contemporaneously, apical organelles in the parasite called rhoptries secrete their proteins and lipid contents to establish the nascent parasitophorous vacuole membrane that surrounds the newly invaded malaria parasite and provides the space into which the invading parasite can move [4].Once the merozoite is inside the red blood cell, the erythrocyte membrane is sealed behind it completing invasion. Remarkably, the invasion process is accomplished within a few minutes [2].

In *P*. *falciparum* two gene families encode important proteins that function in invasion: the erythrocyte binding-like antigens (EBLs) (EBA-140/BAEBL, EBA-175, EBA-181/JESEBL, EBL-1) and reticulocyte binding-like homolog proteins (RBPs or PfRhs) (PfRh1, PfRh2a, PfRh2b, PfRh4 and PfRh5) (reviewed in [1,5,6]). During invasion these ligands are localized at the apical tip of the merozoite and are able to bind erythrocytes. For *P*. *falciparum*, there are several major events that occur as parasite erythrocyte binding proteins engage their respective host erythrocyte receptors. Upon rupture from an infected schizont, merozoites experience a rise in cytoplasmic calcium perhaps as a response to a decrease in potassium ion concentration and exposure of parasites to the extracellular milieu of the blood [7,8]. This results in the release of micronemal proteins such as EBA-175 onto the merozoite surface, which binds to glycophorin A on the erythrocyte surface [8]. This receptor-adhesin interaction restores cytoplasmic calcium levels and triggers the release of rhoptry contents [7,8].

Other studies analysing the hierarchy and coordination of the molecular steps for merozoite invasion using super-resolution microscopy and specific *Pfrh* and *ebl* gene knockouts in *P*. *falciparum* have provided a means to isolate the function of EBA-175 and PfRh4 [4]. These studies have shown that EBA-175 and PfRh4 play a direct role in attachment, subsequently followed by tight junction formation and rhoptry release. Also, there is evidence that the EBL and PfRh protein families mediate attachment to the erythrocyte and initiate an internal signal within the merozoite which triggers release of the rhoptry contents for establishment of the parasitophorous vacuole as the invading parasite moves into the host cell using force generated by the actin-myosin motor [4].

How the parasite communicates a signal from its extracellular binding domain to the molecular machinery within the parasites is yet to be understood. Studies on the cytoplasmic tail of *P*. *falciparum* Apical Membrane Antigen-1 (PfAMA-1) clearly show that phosphorylation of the cytoplasmic tail by Protein Kinase A is essential for parasite invasion [9–11]. However, mounting evidence suggests an important role for the small cytoplasmic domains (also termed tails) within EBL and PfRh proteins for the completion of the invasion process. First, removal of the cytoplasmic domain of EBA-175 results in an inability of *P*. *falciparum* to invade using the EBA-175-glycophorin A receptor-ligand interaction, although its subcellular localization and binding capabilities remain unchanged [12]. Second, PfRh2a/2b chimeric strains showed that the differential ability to use distinct PfRh2a or PfRh2b pathways is conferred by the cytoplasmic domains of PfRh2a and PfRh2b, not by their ectodomain or transmembrane regions [13]. More recently, phosphorylation of Ser^3233^ of the PfRh2b cytoplasmic tail was detected in late stage *P*. *falciparum* parasites although mutation of this site did not have an effect in parasite invasion [14].The acidic regions of the PfRh1, PfRh4 and PfRh2b cytoplasmic tails have been suggested to bind aldolase and glyceraldehyde-3-phosphate dehydrogenase (GAPDH), two proteins known to interact with parasite actin [15]. Thus it has been hypothesized that these interactions may form a bridge between parasite adhesins and the actin-myosin motor. In *Toxoplasma*, it should be noted that the interaction of GAPDH and aldolase with adhesin tails are not required for host cell invasion but rather for parasite metabolism [16]. Therefore, a detailed molecular study is required to determine exact residues and regions within EBL and PfRh cytoplasmic tails that are functionally required for signal transduction during parasite invasion.

In this work, using PfRh4 as a model adhesin, we address the role of the cytoplasmic domain in merozoite invasion. We show that several residues in the PfRh4 cytoplasmic tail serve as substrates for phosphorylation and provide evidence implicating *P*. *falciparum* Casein Kinase 2 (PfCK2). These residues in the PfRh4 cytoplasmic tail are essential for erythrocyte invasion via the PfRh4-CR1 receptor-ligand interaction. This shows that specific residues within cytoplasmic domains are required for successful invasion of malaria parasites into human erythrocytes.

## Results

### Cytoplasmic domains of *P*. *falciparum* adhesins are phosphorylated by parasite kinases

**Fig 1:**
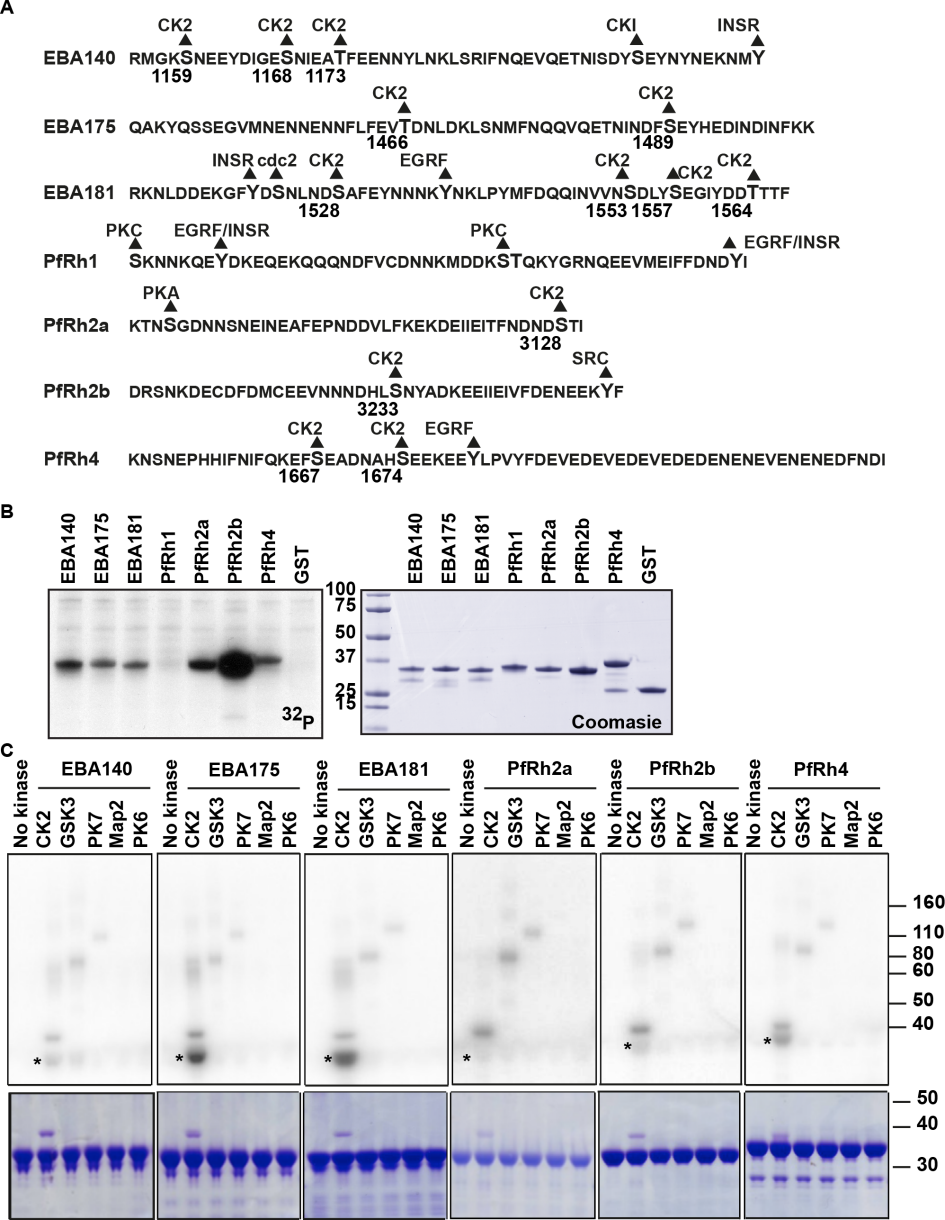
The cytoplasmic tails of *P*. *falciparum* adhesins are phosphorylated *in vitro*. (A) The cytoplasmic tail sequences of the EBL and PfRh protein families are shown. Putative phosphorylation sites for kinases as predicted by NetPhosK are shown with the predicted kinase above the residue and represent the prediction score of the algorithm above 0.5. Residues that have a predicted CK2 site are labelled with the corresponding amino acid number. (B) *In vitro* kinase assays of EBL and PfRh cytoplasmic tails with merozoite lysates. Autoradiograph of *in vitro* kinase assays ^32^P with EBL and PfRh cytoplasmic tail sequences fused to GST. Merozoite lysates were used as a source of kinase activity and the coomasie brilliant blue stained gel of the individual fusion proteins is shown in the right panel. All proteins show labelling with radioactive phosphate except GST alone and the PfRh1 cytoplasmic tail. Molecular weight markers in kDa are shown on the left of the gel. (C) *In vitro* kinase assays of EBL cytoplasmic tails with a small panel of *P*. *falciparum* recombinant kinases. The first lane has only the recombinant cytoplasmic tail whereas subsequent lanes have both recombinant tail and kinase as labelled. Some of the EBL and PfRh tails are phosphorylated by PfCK2 but not by any other kinases in the panel. Migration of each recombinant protein is highlighted with an asterisk.

The cytoplasmic tails of the EBLs and PfRh protein families have several potential phosphorylation sites as determined by the use of the prediction algorithm NetPhosK developed for eukaryotic kinases (Fig 1A) [17]. Strikingly, most of these cytoplasmic tails with the exception of PfRh1 have either single or multiple predicted sites for the acidophilic kinase CK2 (residues highlighted in Fig 1A). To determine if these cytoplasmic tails could be phosphorylated, the tail domains were fused to gluthathione-S transferase (GST) and *in vitro* kinase assays were performed using *P*. *falciparum* D10 merozoite lysate as a source of parasite kinases. All cytoplasmic tails of the EBLs and PfRh proteins were phosphorylated under these conditions with the exception of the PfRh1 (Fig 1B). Control reactions using GST alone showed that the affinity tag had no sites that were recognized by parasite kinases (Fig 1B).

To identify the parasite kinase involved in phosphorylation of these cytoplasmic tails, a panel of active *P*. *falciparum* kinases was used that included PfCK2α, PfGSK3, PfPK7, PfMap2 and PfPK6, in *in vitro* kinase assays with the adhesin cytoplasmic tails. The first lane of these kinase assays contains the labelled cytoplasmic domain without any added kinases (no kinase lane, Fig 1C). The kinases above except for PfMap2 show robust auto-phosphorylation or phosphorylation of known substrates indicative for active kinase activity (S1 Fig). PfCK2 was able to phosphorylate the cytoplasmic domains of PfRh2b, PfRh4, EBA140, EBA175 and EBA181 whereas the other kinases showed significantly less levels of phosphorylation (Fig 1C). Whilst we cannot rule out contributions from other kinases these results suggest that PfCK2 is a candidate kinase for phosphorylation of the EBL and PfRh cytoplasmic domains.

### Site and kinase specificity for PfRh4 cytoplasmic tail phosphorylation

**Fig 2:**
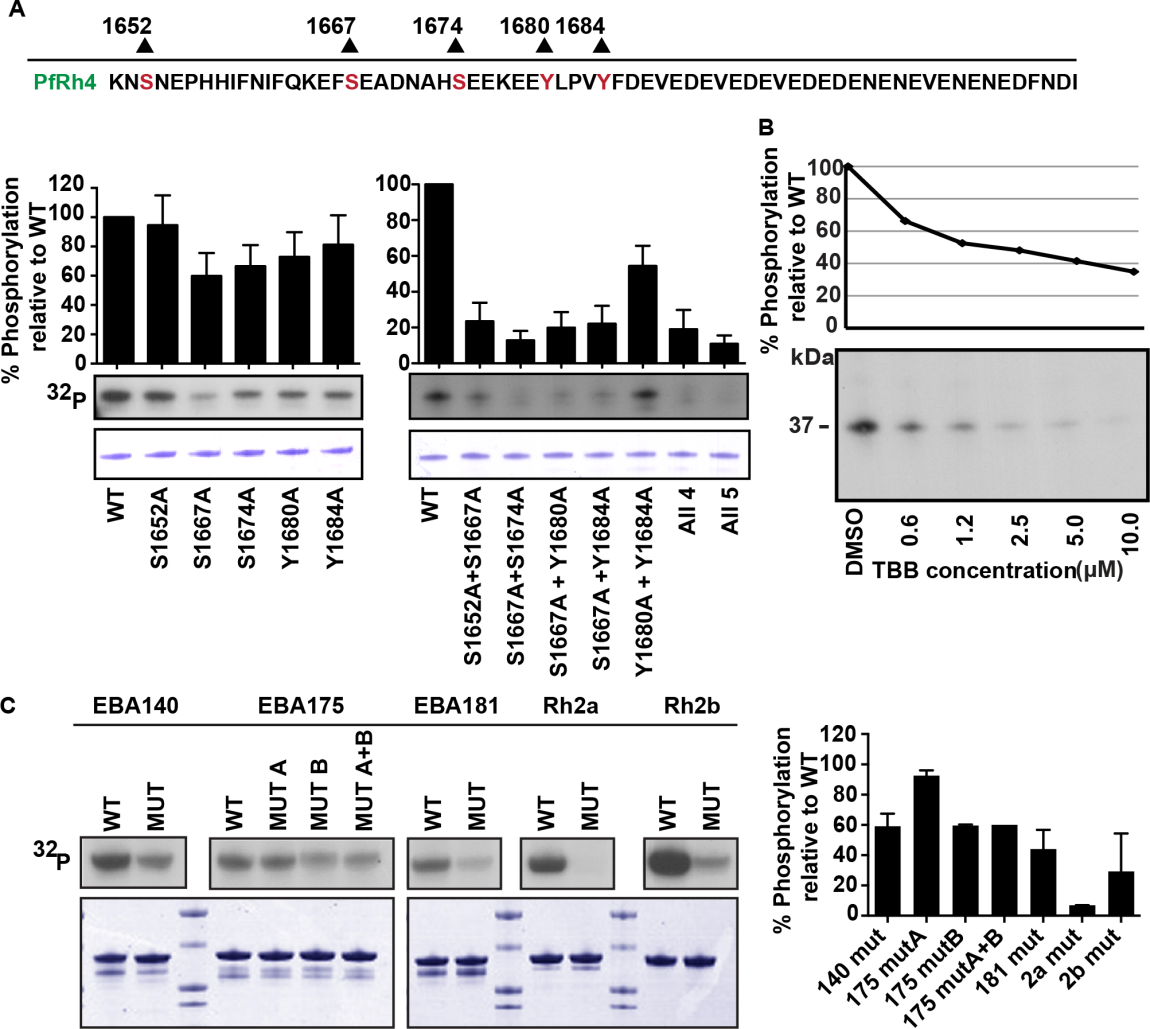
Identification of putative phosphosites and parasite kinase involved in modification of PfRh4 cytoplasmic tail. (A) *In vitro* kinase assays of wildtype and putative phosphosite mutations in PfRh4 cytoplasmic domains. The amino acid sequence of the PfRh4 cytoplasmic tail is shown with serines and tyrosines highlighted with the residue number. Each potential phosphosite on the PfRh4 tail was individually mutated to alanine. The all 4 lane has mutations in S1667A, S1674A, Y1680A and Y1684A and the all 5 lane has S1652A, S1667A, S1674A, Y1680A and Y1684A putative kinase sites mutated. The phosphorylation signal was quantitated and adjusted for protein loading. The loading-adjusted mutant phosphorylation signals were divided by the wildtype and plotted as a percentage of the wildtype signal (Y-axis). Autoradiograph of proteins after incubation in the *in vitro* phosphorylation assay and Coomassie gel from which protein loading was quantitated are shown in lower panels. Lane labels (X-axis) denote residues mutated to alanine. Mean percentage of wildtype phosphorylation +1 standard error of the mean are displayed. Data was averaged from four experiments performed on separate days. (B) Dosage-response curve for PfRh4 tail phosphorylation by merozoite lysate in the presence of increasing concentrations of the CK2 inhibitor TBB. PfRh4 tail phosphorylation was quantitated after incubation in *in vitro* phosphorylation assay with TBB. The phosphorylation signal for each condition was adjusted to reflect the average amount of protein loaded across each condition, determined by densitometry of the Coomassie brilliant blue stained gel. Y-axis represents loading-adjusted phosphorylation signal as a percentage of phosphorylation in the presence of DMSO (control). Autoradiograph of wildtype GST-fused PfRh4 proteins after incubation in the *in vitro* phosphorylation assay. X-axis indicates the TBB concentration with which the phosphorylation assay was incubated or DMSO. (C) *In vitro* kinase assays of PfRh and EBL cytoplasmic tails. The phosphorylation signal was quantitated and adjusted for protein loading. Autoradiograph of proteins after incubation in the *in vitro* phosphorylation assay and Coomassie brilliant blue stained gel from which protein loading was quantitated are shown. Data was averaged from four experiments performed on separate days (right panel) and standard error of the mean is shown. The following sites were mutated: EBA140 (S1159A, S1168A, T1173A), EBA175 (T1466A, mut A) and (S1489A, mut B) and in combination (mut A and B), EBA181 (S1528A, S1553A, S1557A, T1564A), PfRh2a (S3128A) and PfRh2b (S3233).

The cytoplasmic domain of PfRh4 was used as a model adhesin to determine the amino acid residues involved in phosphorylation. This domain has three serine and two tyrosine residues that are potential sites for kinase phosphorylation (Fig 2A). Each serine and tyrosine was mutated individually to alanine within the context of the PfRh4 tail and *in vitro* phosphorylation assays were performed with merozoite lysates as a source of kinase activity. After adjusting for protein loading of each recombinant fragment fused to GST, the ^32^P signal of the mutants was normalized to wildtype PfRh4 tail phosphorylation signal, which was arbitrarily set to 100%. Mutation of serine 1652 to alanine (S1652A) had a negligible effect (Fig 2A, left panel). Mutation of serine 1667 (S1667A) showed 60.0 ± 15.62% of phosphorylation relative to wildtype, however this reduction did not reach statistical significance. Mutation of serine 1674 (S1674), tyrosine 1680 (Y1680) and tyrosine 1684 (Y1684) to alanine resulted in signals at 66.6 ±14.44%, 72.94 ± 16.81% and 81.21 ± 20.11% of the wildtype cytoplasmic domain respectively (Fig 2A, left panel). As no single residue resulted in a significant or complete loss of phosphorylation when mutated to alanine, it is clear that more than one residue contributes to the full level of phosphorylation within the PfRh4 cytoplasmic domain.

To determine which amino acid residues were substrates for phosphorylation, cytoplasmic domains were made that contained multiple mutations of the candidate amino acid residues. Double and multiple mutants were constructed with the S1667A mutation as this mutation showed the highest reduction in phosphorylation in the single mutant analyses. Using *in vitro* kinase assays with parasite lysate, each double mutant combination had a statistically significant effect on the level of phosphorylation (Fig 2A, right panel). In addition, mutation of any putative kinase site in combination with S1667A resulted in a similar reduction in phosphorylation to that observed when either the four (All 4 lane: S1667A, S1674A, Y1680A and Y1684A) or five (All 5 lane: S1652A, S1667A, S1674A, Y1680A and Y1684A) putative kinase sites were mutated. Although the tyrosine residues did not appear to contribute to PfRh4 phosphorylation when mutated singly, mutation of both resulted in a 49.5 ± 6.5% level of phosphorylation relative to wildtype (Fig 2A, right panel).

To further demonstrate the involvement of PfCK2 in phosphorylating the cytoplasmic domain of PfRh4, the specific inhibitor 4,5,6,7-tetrabromo-1H-benzotriazole (TBB) was tested for its effect on phosphorylation (Fig 2B) [18]. Using concentrations of TBB ranging from 0.625 to 10 μM, we observed a dose-dependent decrease in PfRh4 cytoplasmic tail phosphorylation (Fig 2B). We note however, that the specificity of TBB has not been tested in *P*. *falciparum* lysates and may inhibit other parasite kinases apart from PfCK2. In addition, we mutated all predicted PfCK2 phosphorylation sites in the other EBL and PfRh cytoplasmic tails and performed *in vitro* kinase assays with parasite lysate as a source of kinases. The following sites were mutated: EBA140 (S1159A, S1168A, T1173A), EBA175 (T1466A as mut A) and (S1489A as mut B) and in combination (mut A and B), EBA181 (S1528A, S1553A, S1557A, T1564A), PfRh2a (S3128A) and PfRh2b (S3233, residues are highlighted in Fig 1A). In all cases the mutations resulted in a visible reduction in phosphorylation (Fig 2C). Whilst these results suggest that PfCK2 is an important kinase, it appears that other kinases and putative sites are also involved in the phosphorylation of EBA140, EBA175, EBA181 and PfRh2b tails as in most cases, phosphorylation was reduced by no greater that 50% following mutation of predicted CK2 phosphorylation sites (Fig 2C).

### The loss of PfCK2 affects parasite growth

**Fig 3:**
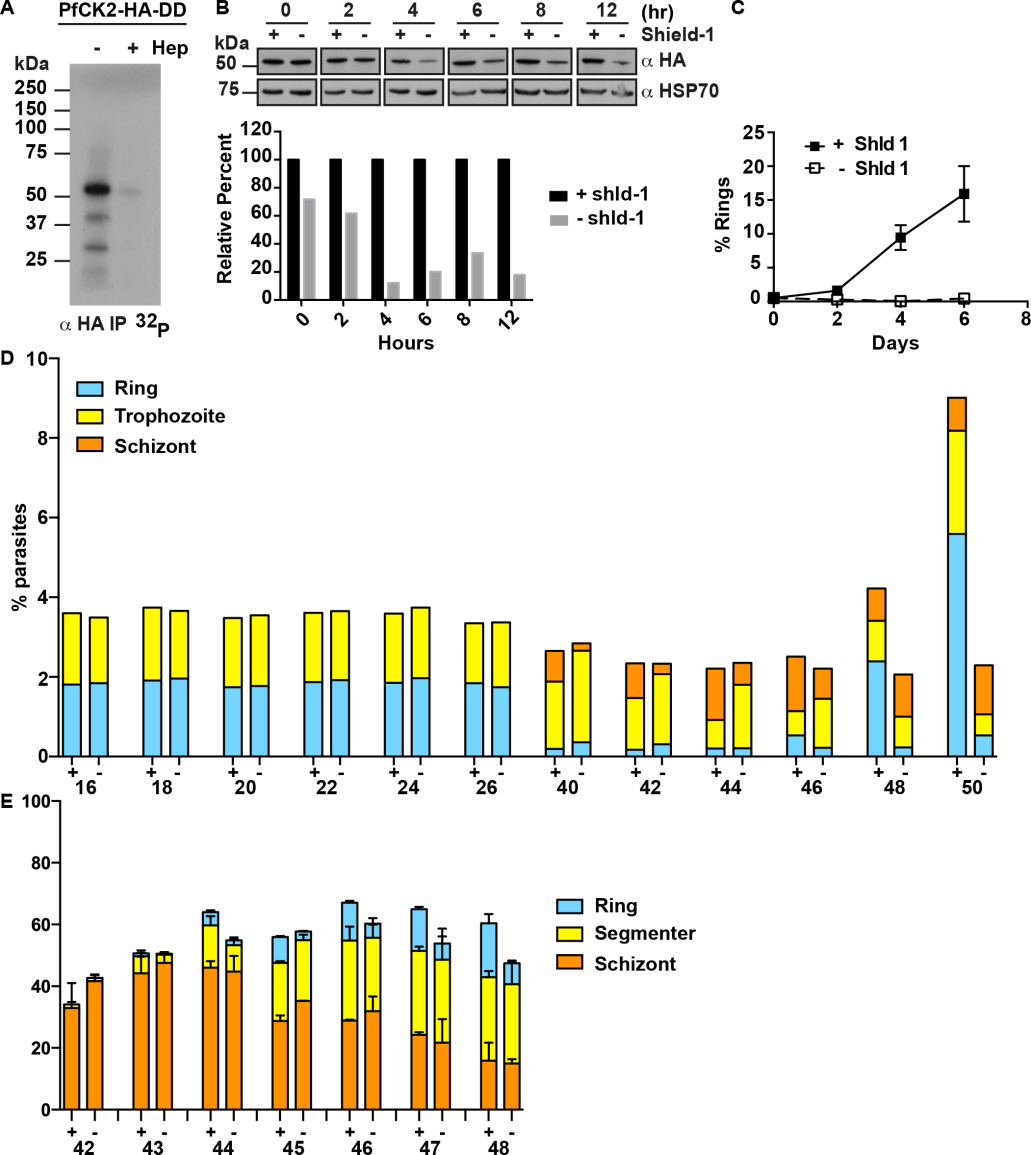
The effect of PfCK2 knockdown on PfRh4 phosphorylation. (A) PfCK2-HA-DD retains kinase activity. PfCK2-HA-DD was immuno-precipitated from parasite lysate using an anti-HA antibody. *In vitro* kinase assay was performed using the immune-precipitatied eluate with the absence or presence of heparin. (B) Time course PfCK2 expression in 3D7-PfCK2DD parasites in the presence and absence of Shield-1. Shield-1 was removed at the ring stage of a synchronous parasite population, Day 0 (5% parasitemia). Absence of Shield-1 resulted in the progressive down-regulation of PfCK2. Parasite proteins were extracted using saponin lysis and analysed by western blotting for the presence of HA-tagged PfCK2 protein. HSP70 served as a loading control for normalisation. Densitometry analysis was performed and shown in bottom graph. (C) Growth assay of PfCK2-HA-DD in the presence and absence of Shield-1 (n = 2, error bars are standard error of the mean (SEM). Identical experiments were performed on a second cloned parasite line and similar results were obtained (n = 2). Parasitemia was quantified by counting Giemsa-stained smears. (D) Progression of PfCK2-HA-DD from late schizont to ring formation with and without Shield-1. Distinct ring, trophozoite and schizont populations were monitored using Sybr Green staining as the parasite progressed from 44 to 50 hours. One representative experiment is shown and was repeated 3 independent times. (E) Progression of PfCK2-HA-DD from late schizont to ring formation with and without Shield-1. Distinct schizont, late segmenter and ring populations were monitored using light microscopy and Giemsa-stained slides as the parasite progressed hourly from 42 to 48 hours. This experiment was performed independently twice and error bars shown are standard error of the mean (SEM).

To examine if the loss of PfCK2 function in parasites would result in a reduction in parasite growth, the destabilization domain (DD) system was used to regulate the amount of PfCK2 in parasites [19]. We genetically fused DD and three hemaglutinin (HA) epitope tags to the 3’ end of the *pfck2α* gene in *P*. *falciparum* and successfully generated clonal lines of PfCK2-HA-DD (S2 Fig). PfCK2-HA expressed by PfCK2-HA-DD parasites retained kinase activity as it was capable of auto-phosphorylation and was inhibited by heparin, also a known CK2 inhibitor (Fig 3A). In the presence of the ligand Shield-1, there was expression of PfCK2-HA-DD, however, upon removal of Shield-1 there was a progressive degradation of PfCK2-HA-DD over time with a maximum degradation of approximately 80% within 4 hours, which did not decrease over longer time points (Figs 3B and S2)

To determine if the function of PfCK2 was essential, growth assays of PfCK2-HA-DD parasites were performed in the presence and absence of Shield-1. In the presence of Shield-1, PfCK2-HA-DD parasites showed increasing parasitemia, however, in its absence, no increase in parasitemia was observed even after 6 days (Fig 3C). Using a shorter time course, parasite progression was followed through the later stages of the asexual life cycle by staining with the DNA stain Sybr Green (Fig 3D). Parasite growth in the presence or absence of Shield-1 did not show any difference in the proportion of rings maturing into trophozoites and schizonts from 16 to 44 hours of the blood stage cycle. Also, from 44 to 50 hours, in the presence of Shield-1, PfCK2-HA-DD parasites were able to rupture and reinvade new erythrocytes (Fig 3D). However in the absence of Shield-1, although there was a distinct mature parasite population, there was not a significant increase in the ring population from 44 to 50 hours (Fig 3D). These results suggest that the decrease in PfCK2 levels caused a defect in merozoite invasion or a delay in parasite progression.

To examine if the decrease in PfCK2 levels would also result in a defect in parasite egress, we removed Shield-1 from trophozoite stage parasites and observed parasites every hour from 42 to 48 hours. Using light microscopy and Giemsa-stained parasite smears, we quantitated the percentage of schizont stage parasites, fully segmented parasites and newly-invaded rings. We observed that PfCK2 knockdown parasites were still able to generate rings which suggest that that late segmented parasites are still able to egress and invade fresh erythrocytes (Fig 3E).

### The cytoplasmic tail of PfRh4 is required for *P*. *falciparum* merozoite invasion

**Fig 4:**
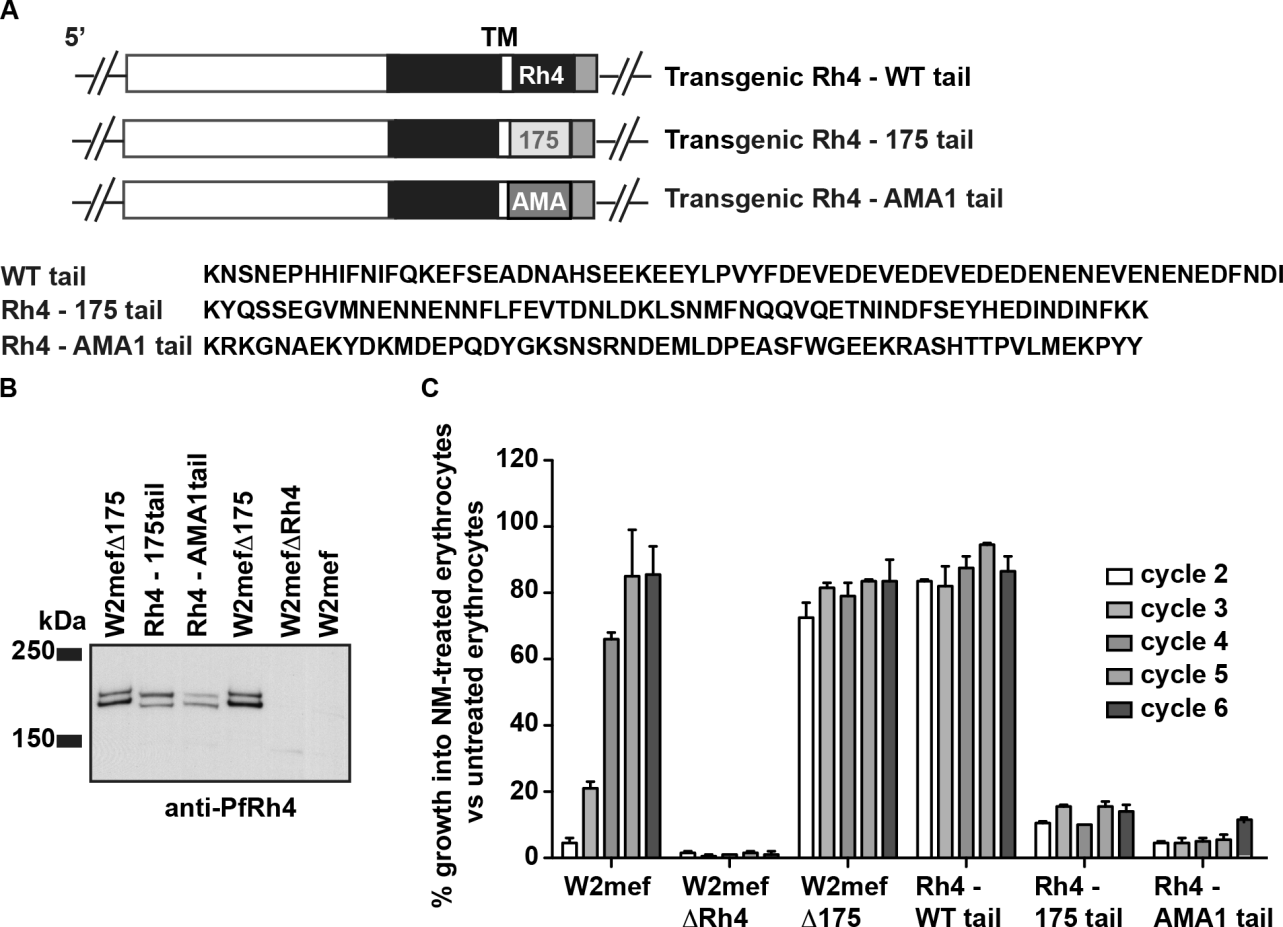
Substitution of the PfRh4 cytoplasmic tail results in a defect in merozoite invasion. (A) Schematic of the substitution of PfRh4 cytoplasmic tail with cytoplasmic domains from EBA-175 and AMA1. The amino acid sequence of each respective tail region is shown at the bottom. (B) Expression of the transgenic PfRh4 lines containing a substitution in the cytoplasmic tails. PfRh4 expression was seen in W2mefΔ175 but not in the W2mefΔRh4 or parental W2mef strain as expected. The difference in protein migration between PfRh4-175tail and PfRh4-AMA1 tail is likely due to the difference in acidic residues between the various cytoplasmic tails. The molecular weight marker is labelled on the left of the panel (kDa). (C) Growth assays of the PfRh4 substituted cytoplasmic tail transgenic lines. Parasitaemia was measured in neuraminidase-treated, and untreated erythrocytes after every 48 hours incubation (labelled as cycles). W2mef∆175 and Rh4-WT tail expresses PfRh4 and can invade neuraminidase-treated erythrocytes whereas W2mef∆PfRh4 does not express PfRh4 under these experimental conditions are not able to invade neuraminidase-treated erythrocytes. W2mef shows an ability to invade neuraminidase-treated erythrocytes upon selection on these cells. Both Rh4-175tail and Rh4-AMA1tail were not able to fully grow in neuraminidase-treated erythrocytes. The parasite lines used in this experiment are displayed on the X-axis. The y-axis represents parasitaemia of neuraminidase-treated erythrocytes as a percentage of parasitaemia of the same line grown on untreated erythrocytes. Error bars represent +1 standard error of the mean. Assay performed three times on separate days, each in triplicate.

**Fig 5:**
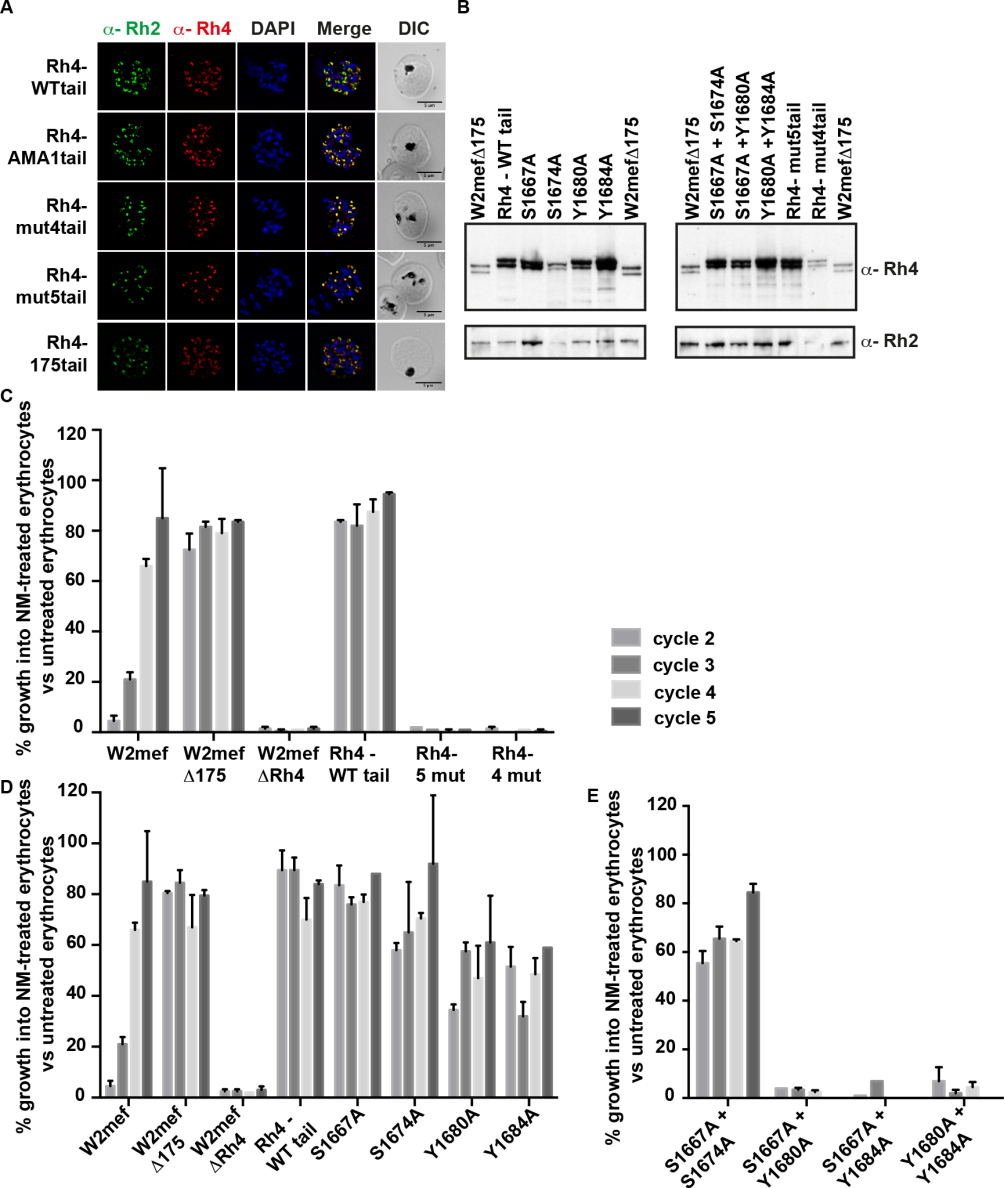
Specific amino acid residues in the cytoplasmic domain are essential for PfRh4 function. (A) Localization of PfRh4 and PfRh2 in Rh4-WT tail, RH4-AMA1tail, Rh4-mut4tail and RH4-mut5tail lines as detected using anti-PfRh4 monoclonal and anti-PfRh2 polyclonal antibodies. Parasite nuclei were stained with DAPI. (B) Expression of PfRh4 and PfRh2 in W2mefΔ175 and all transgenic lines as detected by anti-PfRh4 and anti-PfRh2 antibody. All transgenic lines migrated as a slightly larger doublet compared to PfRh4 in W2mefΔ175, consistent with the addition of the hexa-histidine tag at the C-terminus of the protein. (C) Growth assays of transgenic lines with four and five mutations at serine and tyrosine amino acid residues in the PfRh4 cytoplasmic tail. (D) Growth assays of single mutations in the PfRh4 cytoplasmic tail. (E) Growth assays of double mutations in the PfRh4 cytoplasmic tail. In all three panels, parasitaemia was measured in neuraminidase-treated, and untreated erythrocytes after every 48 hours incubation (labelled as cycles). The parasite lines used in this experiment are displayed on the X-axis. The y-axis represents parasitaemia of neuraminidase-treated erythrocytes as a percentage of parasitaemia of the same line grown on untreated erythrocytes. Error bars represent +1 standard error of the mean. Assay performed three times on separate days, each in triplicate.

The W2mef strain of *P*. *falciparum* is particularly reliant on EBA-175 as a key parasite invasion ligand and the gene encoding PfRh4 is silenced [20–24]. However, loss of EBA175 function, either by *eba175* gene disruption or receptor ablation by treatment of erythrocytes with neuraminidase, results in selection of W2mef parasites that express high levels of PfRh4 [21–23]. This suggests that the function of EBL and PfRh protein families are overlapping and that PfRh4 is capable of complementing the loss of EBA-175 function. To determine if the cytoplasmic domains of these adhesins are functionally equivalent a transgenic line called Rh4-175tail was constructed that substituted 67 amino acids of PfRh4 tail with 60 amino acids of EBA-175 cytoplasmic domain (Fig 4A). Additionally, the PfRh4 tail was replaced with the cytoplasmic sequence of AMA1, generating a line called Rh4-AMA1tail. Both Rh4-175tail and Rh4-AMA1tail expressed the modified PfRh4 (Figs 4B and 5A).

To determine if these mutant PfRh4 chimeric proteins could function in merozoite invasion the transgenic *P*. *falciparum* were tested for their ability to invade neuraminidase-treated erythrocytes. Neuraminidase-treatment of erythrocytes blocks the ability of *P*. *falciparum* merozoites to use ligands that bind to sialic acid-containing receptors. PfRh4 binds to CR1 and this interaction is not affected by neuraminidase-treatment and selection of W2mef parasites results in selection for those in which the *pfrh4* gene has been activated and functional [25–27]. Growth of the transgenic parasites was monitored over 12 days to determine if the mutant PfRh4 proteins were functional (Fig 4C). The control lines W2mef, W2mefΔRh4 (lacks PfRh4 function), W2mefΔ175 (PfRh4 expression activated) and Rh4-WT tail (tail replaced with the WT Rh4 tail sequence) were also tested. Note that when Rh4-WT tail is integrated the activated transcription at the nearby drug cassette activates the native PfRh4 promoter, which results in transcription of Rh4-WT tail. In wildtype W2mef however, the PfRh4 promoter is not activated in the major of parasites until selection on neuraminidase-treated cells selects for the population of cells that activate transcription of PfRh4. As in previous studies, growing W2mef parasites on neuraminidase-treated erythrocytes results in the selection of a parasite population in which PfRh4 was expressed and functional (Fig 4C). As expected the Rh4-WTtail and W2mefΔ175 parasites were capable of invading neuraminidase-treated erythrocytes (86% and 79% respectively, average value from all growth cycles) because they also express fully functional PfRh4. In contrast, W2mefΔRh4 was not able to invade and grow in neuraminidase-treated erythrocytes (1%, average value from all growth cycles, Fig 4C). The Rh4-EBA175tail and Rh4-AMA1tail parasites showed a dramatic reduction in their ability to grow in neuraminidase-treated erythrocytes (12 and 4% respectively, average value from all growth cycles, Fig 4C). These results show that the amino acid sequence within the PfRh4 cytoplasmic tail was functionally important and could not be complemented by the cytoplasmic domains from a member of the EBL protein family or AMA-1.

### Specific amino acids of the PfRh4 cytoplasmic domain are essential in its function in merozoite invasion

To determine if the putative phosphorylation sites within the PfRh4 tail played a role in parasite growth, transgenic lines were generated with single and multiple amino acid mutations. Initially, parasite lines that expressed mutant PfRh4 were constructed in which all four and five putative serine and tyrosine phosphosites within the cytoplasmic tail were mutated. Rh4-mut4 tails contains the mutations S1667A, S1674A, Y1680A and Y1684A, and Rh4-mut5 tail contains similar mutations with an addition mutation at S1652A within the PfRh4 cytoplasmic tail respectively which migrated as a slightly larger doublet compared to PfRh4 in W2mefΔ175, consistent with the addition of the hexa-histidine tag at the C-terminus of the protein (Fig 5B). Rh4-mut4 tail and Rh4-mut5 tail expressed PfRh4 and all showed an apical localization similar to another rhoptry protein PfRh2 (Fig 5A). The PfRh4 protein was functional in Rh4-WT tail line but not functional in any of the other mutant parasite lines, as they were not able to grow in neuraminidase-treated erythrocytes (~ 1%, Figs 4C and 5C). These results suggest that all or some of these amino acids in the cytoplasmic domain of the 205 kDa PfRh4 play an essential role in merozite invasion.

To determine the importance of specific amino acids in the function of the PfRh4 cytoplasmic tail transgenic lines were generated with single mutations at the position of the serine and tyrosine residues. Despite repeated attempts we were unable to obtain integration of the S1652A mutant. However, the other mutant lines expressed the tagged PfRh4 (Fig 5B). In contrast to the prediction from our *in vitro* kinase assays, mutation of individual serine residues resulted in a very small effect on parasite growth and PfRh4 function (ranging from 81 to 71%, Fig 5D). However, mutation of individual tyrosine residues resulted in a 50% reduction in parasite growth in neuraminidase-treated erythrocytes (Fig 5D). The effect of mutating tyrosine residues within the cytoplasmic tail became more pronounced when combined with S1667A (2–3%) or when both tyrosine residues were mutated within the tail (3.5%) (Fig 5E). However, mutation of both serine residues (S1667A and S1674A) did not show a further reduction in PfRh4 function compared to S1674A alone (67 vs 71% respectively, Fig 5E). These results show that S1667, Y1680 and Y1684 are key amino acids in the PfRh4 cytoplasmic tail for this protein’s function in parasite growth.

### Live imaging of the invasion defect of transgenic *P*. *falciparum* strains harbouring mutations in the PfRh4 cytoplasmic domain

**Fig 6:**
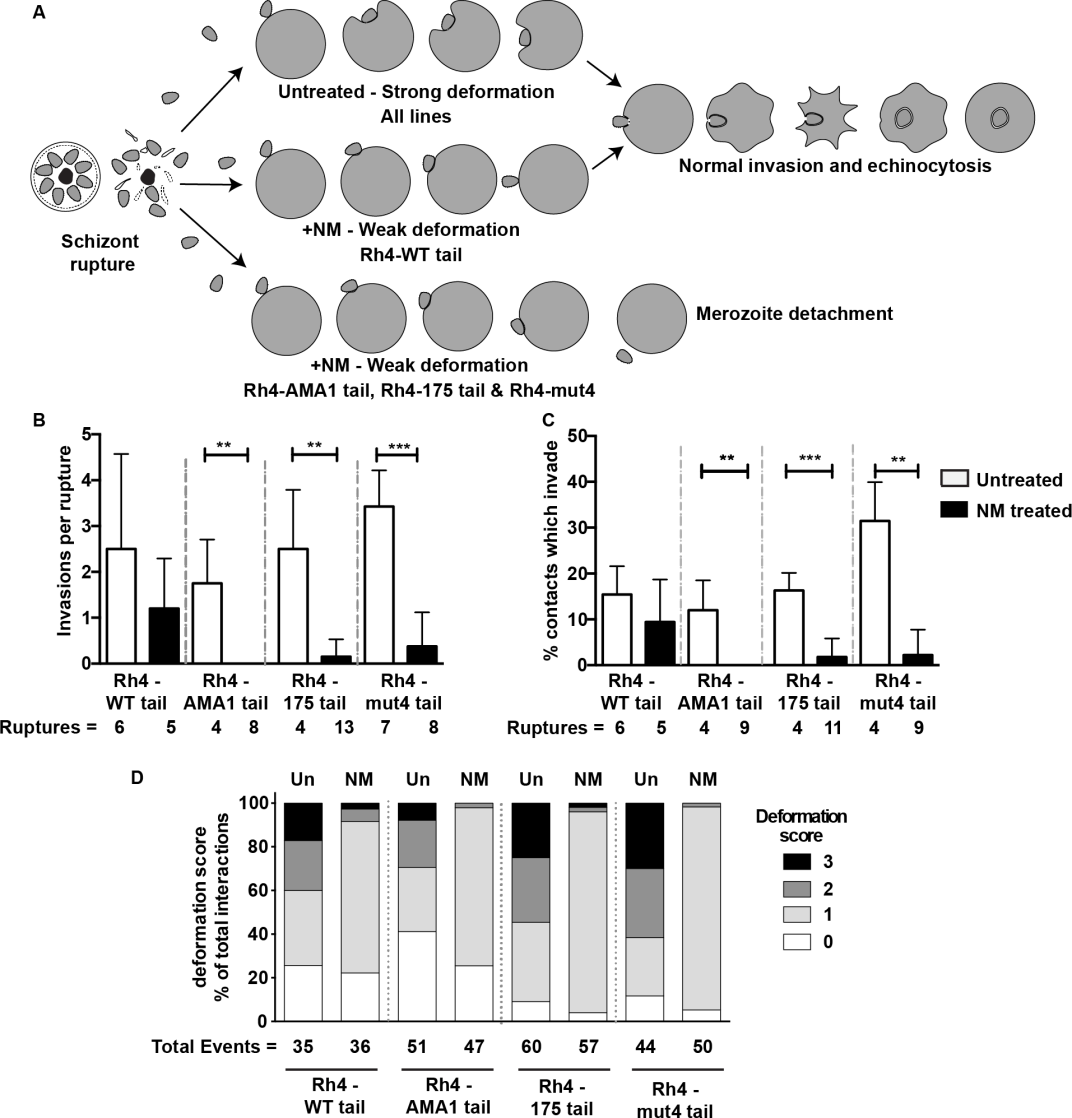
Live Imaging of Rh4-mut tail variant show that parasites are unable to efficiently invade neuramindase—treated erythrocytes. (A) Graphical interpretation of the video microscopy of live merozoites expressing modified PfRh4 tails attempting to invade NM treated and untreated erythrocytes. (B) Merozoite invasions per schizont rupture in NM-treated and untreated erythrocytes. Ruptures = the number of schizont ruptures that were imaged and followed for merozoite invasion. (C) The percentage of merozoite contacts with erythrocytes lasting ≥0.25s that leads to successful invasion. Shown is the average (mean and standard deviation) of the percentages of invasions per number of contacts observed for each schizont rupture. (D) Deformation scores of merozoites contacting erythrocytes in NM-treated and non-treated erythrocytes. Shown are the percentages of each deformation score from the total number of contacts. The total number of events (contact between a merozoite and RBC) counted for each experimental condition is shown. Statistics: B and C, Mann-Whitney test where *p<0.05, **p>0.01, **p<0.01; D, Chi square test.

Our growth assays indicate that transgenic parasites with mutations within their PfRh4 cytoplasmic tail lose the ability to thrive in the presence of neuraminidase-treated red blood cells. We hypothesize that this decline in growth was due to a reduction of successful invasions employing the PfRh4-CR1 invasion pathway. To verify this hypothesis, we performed live imaging of transgenic parasites during invasion to determine how efficiently they invaded and where the process was being blocked. Wild type merozoites contact neighbouring red blood cells and proceed through the stages of pre-invasion, internalization and echinocytosis. PfRh4 is thought to act during pre-invasion when strong deformation and reorientation of the parasite occurs (Fig 6A) [28]. Imaging of merozoites following schizont rupture indicated that Rh4-WT tail, Rh4-mut4 tail, Rh4-AMA1 tail and Rh4-175 tail parasite lines had a mean of 1.5 to 3.5 invasions per rupture (Fig 6B). Following neuraminidase-treatment of erythrocytes, which forces merozoites to utilize the PfRh4-CR1 invasion pathway, the number of successful invasions per rupture declined significantly in Rh4-175 tail and Rh4-mut4 tail lines and no invasions were observed in the Rh4-AMA1 tail line (Fig 6A and 6B). In contrast, there was no significant change in the mean number of invasions per rupture of PfRh4-WTtail parasites into neuraminidase-treated erythrocytes.

Since the number of invasions per rupture was dependent on the density of surrounding red blood cells, which may vary from rupture to rupture, we assessed the percentage of merozoite contacts with erythrocytes that lead to successful invasion for each schizont rupture. Reflective of the analysis above, nearly all lines showed a similar percentage of contacts that resulted in successful invasion, and using neuraminidase-treated erythrocytes, Rh4-mut4 tail, Rh4-AMA1 tail and Rh4-175 tail parasite lines invaded much less efficiently, while the Rh4-WT tail line had no significant decrease in contacts that led to invasion (Fig 6C). Whilst the Rh4-mut4 tail merozoites appeared to exhibit more invasions per rupture and a greater percentage of contacts that invaded compared to the other lines it is likely that this is not significant (Fig 6B and 6D). It is likely that this will differ from experiment to experiment dependent on factors such as parasitaemia and hematocrit. Collectively, these results show that mutation or substitution of the PfRh4 cytoplasmic tail renders merozoites incapable of using the PfRh4-CR1 invasion pathway and directly results in the lack of parasite proliferation in neuraminidase-treated red blood cells.

Since the stage of pre-invasion where PfRh4 is thought to play a role is characterized by strong deformation and reorientation, we analysed how mutation of the PfRh4 tail affected these parameters. We used our previously published four point deformation scale with zero being no deformation and three being strong deformation [28]. Merozoites from Rh4-mut4 tail, Rh4-AMA1 tail and Rh4-175 tail parasite lines showed similar levels of deformation and invasion upon contact with untreated erythrocytes as compared to the Rh4-WT tail (Fig 6D). Following neuraminidase-treatment, however, the deformation scores were much lower for all lines, in keeping with our previously published results (Fig 6D). Merozoites from the Rh4-mut4 tail, Rh4-AMA1 tail and Rh4-175 tail parasite lines attached and often weakly deformed the neuraminidase-treated erythrocyte surface as they moved over it but could rarely penetrate it (Fig 6A) (S1, S2 and S3 Movies respectively). The Rh4-WT tail merozoites were likewise unable to strongly deform neuraminidase-treated erythrocytes but were still able to invade which stands in contrast to previous data positively correlating successful invasion with strong deformation [28] (S4 Movie). Therefore, we show here for the first time, that PfRh4 dependent invasion does not require strong deformation, which implies that EBA proteins interacting with their cognate glycophorin receptors are primarily responsible for robust deformation. Overall these results indicate that PfRh’s and EBA’s likely have complementary functions rather than being completely redundant and that the cytoplasmic tail of PfRh4 plays an important role in triggering downstream events affecting invasion, which are not functionally substituted by the tails of other invasion ligands.

## Discussion

The EBL and PfRh family of adhesins in *P*. *falciparum* parasites mediate recognition of human erythrocytes for invasion [1]. Upon attachment of these proteins to the erythrocyte, current evidence suggests that they also function to signal downstream events within the parasite such as rhoptry secretion and tight junction formation [7,8,13,14]. We have investigated the potential role of these adhesins to signal downstream events in invasion after initial merozoite interaction with the host cell. We have shown that specific amino acids in the cytoplasmic domain of these adhesins, including PfRh4, are substrates for phosphorylation by parasite kinases. PfCK2 has been implicated as an important kinase for phosphorylation of these cytoplasmic domains for signalling of downstream events in merozoite invasion.

PfRh4 was used as a model adhesin to analyse its function in signalling and merozoite invasion as we can block the function of other members of the EBL and PfRh family by treatment of erythrocytes with neuraminidase [20,23]. This enabled us to test the role of the cytoplasmic tail and specific amino acids and show that they were essential for the function of PfRh4 in merozoite invasion of human erythrocytes. The mutant merozoites were able to attach to erythrocytes but they were defective in their ability to deform and invade neuraminidase-treated erythrocytes. These results indicate that specific amino acids, within the cytoplasmic domains of PfRh4 maybe involved in signalling of downstream invasion events.

Our results have shown that the cytoplasmic domains of other *P*. *falciparum* ligands are unable to complement the function of the equivalent region in PfRh4. This is in contrast to previous results that showed substitution of the EBA175 cytoplasmic tail with the C-terminal amino acids of TRAP, a protein involved in sporozoite invasion into liver cells, was fully functional for merozoite invasion [12]. This has suggested that both TRAP and EBA175 cytoplasmic tails have the same function and likely bind to the same adaptor protein in invasion regardless of host cell type. It suggests that PfRh4 and EBA175 may have some distinct functions in merozoite invasion with respect to the role of the cytoplasmic domain. However, disruption of the gene encoding EBA175 in W2mef parasites results in selection of those that have activated expression of PfRh4 suggesting that it is capable of complementing this loss-of-function [23]. This is consistent with other *P*. *falciparum* lines where disruption of the *eba175* gene results in increased transcription of the *pfrh4* gene [29]. Whilst the cytoplasmic domains of EBA175 and PfRh4 appear to be functionally distinct it is possible that the downstream effects in the merozoite may be similar.

One clear difference between PfRh4 and EBA175 cytoplasmic tail may be their ability to recruit aldolase and GAPDH [15]. These studies were done using peptides encompassing only the C-terminal acidic domains of these cytoplasmic regions and do not include most of the putative phosphorylation sites analysed in this study. In particular, the acidic nature of these cytoplasmic domains in combination with the presence of subterminal tyrosine or phenylalanine appear necessary for aldolase binding to the PfRh1, PfRh4 and PfRh2 tails [15]. It has been thought that the binding of the PfRh tails to aldolase and GADPH creates a bridge with the parasite action-myosin motor that drives invasion. However, the acidic domain of EBA175 cytoplasmic domain does not recruit aldolase and this remains one of important differences with respect to PfRh4 and their individual function in parasite invasion. In *Toxoplasma*, it should be noted that the interaction of GAPDH and aldolase with adhesin tails are not required for host cell invasion but rather for parasite metabolism [16].

From our analyses it was clear that tyrosine residues within the PfRh4 tail play a key role in the PfRh4-CR1 invasion pathway and that this likely involves phosphorylation. Classical tyrosine kinases have not been identified in *P*. *falciparum* and the general consensus has been that they do not exist [30]. However, recent analyses of global phospho-proteomes detected several tyrosine phosphorylation sites though many of these resided within the activation loop of kinases (reviewed in [31]. Taking in account a higher false discovery rate for phosphor-tyrosine, it has been calculated that only 0.25–0.5% of the parasite’s phospho-proteome is due to tyrosine phosphorylation. In yeast and in human cells, CK2 has been shown to phosphorylate tyrosine residues on specific substrates [32]. The consensus sequence for phosphorylation by CK2 is S/T-X-X-D/E/pS/pY where X can be any amino acid except proline, though it must be noted that other tyrosine phosphorylation sites that have been detected that do not conform to this canonical sequence. Of note is that the tyrosine residues within PfRh4 cytoplasmic tails sit within an extremely acidic region that is conducive for CK2-mediated phosphorylation. Studies of yeast Fpr3 show that CK2-mediated phosphorylation of a tyrosine within an acidic region requires preliminary phosphorylation of a proximal serine [33,34]. It may be the case that PfCK2 initially phosphorylates the serine residues within the PfRh4 cytoplasmic tail. This signal may “prime” the cytoplasmic tail for the necessary phosphorylation of the tyrosine residues when the PfRh4 engages with its receptor CR1 and thus commits the parasite to invasion. This hypothesis is consistent with the contribution of multiple amino acids to the overall level of phosphorylation.

Mutation of the putative phosphosites within the PfRh4 cytoplasmic tail resulted in a loss of phosphorylation and also of the ability of the merozoite to invade neuraminidase-treated erythrocytes. This is consistent with phosphorylation of these amino acids playing an essential role in signaling downstream event after the engagement of PfRh4 with its receptor CR1. However, in most cases, the loss of phosphorylation did not correlate with the extent to which the PfRh4 function was affected for invasion. The effect of the mutation(s) in the PfRh4 cytoplasmic tail on parasite growth is the most relevant measure of the functional importance of each residue. PfRh4 is a type I membrane protein and the proximity of the potential phosphosites to the membrane may be important in regulating signaling events during invasion and how accessible the cytoplasmic tail might be to interacting proteins. One may envision that binding of PfRh4 to CR1 transduces a signal from the extracellular binding domain to the cytoplasmic domain resulting in a change in conformation within the cytoplasmic side of the membrane. This may facilitate subsequent phosphorylation of the tail, which then allows recruitment of the relevant proteins for the downstream events in invasion. The mechanism as to how the extracellular binding of the receptor CR1 initiates transfer of information to the cytoplasmic tail of PfRh4 remains to be investigated

It is interesting to highlight that another common post-translational modification is tyrosine O sulfation [35]. In eukaryotic systems, sulfated tyrosine assist in the enhancement of protein-protein interactions. There has not been any report identifying tyrosine O sulfation of proteins in *P*. *falciparum* and genome searches suggest that this parasite lacks sulfotransferases and other enzymes required for synthesis of the necessary co-factors. Therefore it is unlikely that this potential modification of tyrosine residues occurs and is relevant.

Video imaging of live *P*. *falciparum* merozoites invading human erythrocytes has provided important information on the mechanics of this process and the function of specific proteins at each step [28]. Performing live imaging on lines with specific mutations in PfRh4 has provided information of the role of this protein during the different stages of invasion. In the absence of PfRh4 function the mutant merozoites are unable to invade neuraminidase-treated erythrocytes but still contact and maintain attachment to the host cell. It has been shown that engagement of EBA175 to glycophorin A results in the release of rhoptry proteins [7,8]. In the case of PfRh4, perhaps its interaction with CR1 results in the activation/release of other parasite factors at the apical tip. These factors may still reside within the parasite and respond only to appropriate signaling by a functional PfRh4 cytoplasmic tail. One possibility is the release of rhoptry bulb contents such as lipids or proteins that assist in the establishment of the new parasitophorous vacuole in which the parasite resides. These lipids and proteins may have some ability to modulate the erythrocyte cytoskeleton.

This work has shown that the cytoplasmic tail plays an essential role in the function of PfRh4 in *P*. *falciparum* merozoite invasion. Our current evidence suggests that this involves the phosphorylation of key amino acids in these domains, and that this provides a signal for subsequent downstream events for merozoite invasion. An objective for future work is to identify the adaptor proteins that may bind to the cytoplasmic domains of these proteins, especially in a phosphorylation-dependent manner and transfer the signal for activation of the invasion process. It is also of interest that the EBA175 cytoplasmic tail may recruit different parasite factors compared to PfRh4 and future work will need to address the multiple complexes that signal during this rapid phase in invasion.

## Materials and Methods

### Phosphorylation site and kinase prediction

*P*. *falciparum* EBL and PfRh adhesin family tail domains were defined as residues C-terminal to their respective TM domains which are previously published (Baker et al., 2006). Tail sequences were retrieved from the PlasmoDB database (http://plasmodb.org/plasmo/) and entered into the NetPhos v2.0 (www.cbs.dtu.dk/services/NetPhos/) search engine to determine serine/threonine/tyrosine sites (phosphosites) likely to be phosphorylated (Blom et al., 1999). Sequences were submitted to NetPhosK v1.0 (www.cbs.dtu.dk/services/NetPhosK/) to predict site-specific kinases for adhesin tail sequence [17].

### DNA cloning

For the GST fusion protein constructs, we retrieved the PfRh4 cytoplasmic tail sequence (201 bp) from the *P*. *falciparum* genome database PlasmoDB (www.plasmodb.org). This region was gene synthesized (GenScript) with flanking *Bam*HI and *Xho*I restriction sites and cloned in the plasmid puc57 to generate PfRh4puc57. PfRh4puc57 was digested with *Bam*H1 and *Xho*1 and ligated into the corresponding *Bam*HI-*Xho*I site of PGEX4T-1 vector (GE Healthcare) encoding an amino terminus GST tag. Positive clones identified by restriction digest and confirmed by sequencing. All PfRh4 phosphosite, tail-swap, and tail sequences of the EBA and PfRh adhesins were synthesized (GenScript) and cloned as described above.

Wildtype and mutant PfRh4 tail constructs for 3’ integration into the endogenous PfRh4 gene were created using a previously published integration plasmid hhRh4 [36]. The hhRh4 plasmid comprised ~800 bp of the 3’ PfRh4 sequence followed by a hexa-histidine tag, a terminator sequence (PbT1), and *hdhfr* gene under the control of the *P*. *falciparum* calmodulin promoter to confer resistance to the anti-malarial drug WR99210. Synthetic gene fragments of the PfRh4 cytoplasmic domain were ordered in puc57 (GenScript) encoding alanine replacement of a single serine or tyrosine residue on the PfRh4 tail. Two ‘tail-swap’ constructs encoding replacement of the PfRh4 cytoplasmic domain with the tail of EBA175 or AMA1 were also ordered. All synthetic genes were flanked by *Bsm*1 and *Pst*1 restriction sites, and the corresponding restriction enzymes were used digest the original hhRh4 plasmid and synthetic fragments before gel purification (QiaQuick Gel Extraction Kit, Qiagen). Purified products were ligated, and transformed into STBL4 cells (Invitrogen). Positive transformants were confirmed by restriction digest and sequencing. Positive clones were grown in 600 ml LB and DNA purified for *P*. *falciparum* transfection using a Maxiprep protocol (Qiagen).

### Recombinant protein purification

All GST-fused adhesin tails, and the α subunit of *Pf* protein kinase CK2 (PfCK2α) [37] were expressed as follows. 600 ml of LB was inoculated with a 3 mL overnight culture grown at 37°C. Flasks were incubated for 2 hr at 37°C, and then induced with 0.42 mM IPTG (Sigma) followed by a 5 hr incubation at 30°C, and subsequently harvested. Pellets were resuspended in 50 mL HTPBS with 1 mM EDTA, 5 mM DTT, 1x10^6^ U lysozyme (MP Biomedicals), and protease inhibitor (Roche), and rotated at 4°C for 2 hr. The bacterial suspension was sonicated for 6 x 20 secs and incubated in 1% (v/v) TX100 (Sigma) for 15 mins at 4°C. Insoluble material was pelleted at 10,000 RPM for 30 mins. The supernatant was removed and incubated overnight at 4°C with glutathione-agarose beads (Sigma). Beads were washed in protein purification wash buffer (3x 10 mins, 4°C). GST-fused protein was eluted using glutathione elution buffer in three 1 mL fractions. The eluate was dialysed in 25 mM TrisHCl (pH 7.5) overnight and stored in 20% (v/v) glycerol (Univar) at -80°C until required.

### In vitro phosphorylation assay

*In vitro* phosphorylation reaction comprised of the immobilized fusion protein incubated with 5 μL merozoite lysate (as a source of *P*. *falciparum* kinases), 4.5 μCi γ-^32^P ATP (Perkin Elmer), and 25 mM MgCl_2_ in Buffer B [9]. Merozoite lysate was prepared as previously described [9,38]. For kinase assays using PfCK2, 2 μM recombinant PfCK2α was used instead rather than merozoite lysate. To immobilise the fusion proteins, 40 μg of recombinant protein was bound to 100 μl glutathione-agarose beads overnight at 4°C. For each experiment, 30 μL of protein-bound beads were used. Kinase reactions were performed for 30 mins at 37°C. Beads were washed extensively with Buffer B, and GST fused recombinant protein eluted by boiling in reducing sample buffer. Eluted proteins were separated by SDS-PAGE (4–12% Bis-Tris gel, MES buffer) and stained with Coomassie Blue or transferred to a PVDF membrane (Invitrogen).

Quantitation of phosphorylation levels relative to protein concentration was performed in the following manner. Densitometry measured incorporation of ^32^P into fusion proteins (Fuji FLA-3000 scanner, Image Gauge v4 software, FUJIFILM), and protein density on the corresponding Coomassie loading control gel (Bio-rad GS-800, Quantity One software, Biorad). To correct for signal differences resulting from loading error, mutant density was divided by WT density to give an adjustment factor (set to 1 for the WT), which was multiplied by the ^32^P signal of the corresponding protein. Paired one-tailed t-tests thresholded at p = 0.05 were then performed to compare each mutant to the WT. To represent mutant phosphorylation levels as a percentage of WT, the loading-adjusted signal of each mutant was divided by the WT signal (Fig 2A, 2B and 2C). Each experiment was repeated at least three times on different days. For final Figs, PVDF membranes were exposed to X-ray film (GE Healthcare).

For EBL and PfRh tail phosphorylation assays, and kinase inhibition assays, the ^32^P signal was corrected according to the average amount of protein loaded across the experiment, as determined by Coomassie densitometry.

### Indirect immunofluorescence assay (IFA)

Late-stage schizonts were enriched using a magnet as described previously [38]. Parasites were smeared onto glass slides and fixed with 100% ice-cold methanol for 30 seconds. Slides were blocked overnight in 3% BSA (Sigma) in PBS at 4°C. Diluted primary antibodies were applied to the slides and incubated for one hour at room temperature. The slides were washed three times with PBS and diluted secondary antibodies were applied and allowed to incubate for one hour at room temperature protected from light. After washing the slides three times in PBS, they were mounted in VectaShield (Vector Laboratories) with 0.1 ng/μl 4’,6-diamidino-2-phenylindole (DAPI, Invitrogen). Primary antibodies were used at the following concentrations: mouse anti-PfRh4 (2 mg/ml, 1:500), rabbit anti-Rh2 (2 mg/ml, 1:500). Secondary antibodies were used at the following concentrations: Alexa Fluor 488 goat anti-mouse (1:500) and Alex Flour 594 goat anti-rabbit (1:500). IFA images were obtained using DeltaVision Elite widefield fluorescence microscope. Z stacks processed using Axiovision deconvolution software package.

### Parasite culture and transfection

Parasite transfection was performed as described [36]. 100 μg purified DNA was ethanol precipitated and resuspended in 15 μl of 1XTE. 385 μL of prewarmed Cytomix (Wellems, Fidock) was added to the DNA solution. This solution was mixed with a 5 mL pellet of W2mef-infected erythrocytes and transferred to a 2 mm gap cuvette (GenePulser) for electroporation (0.31 kV/950 uF at infinite resistance) (GenePulser II, Bio-rad). The sample was added to 10 mL 3% haematocrit. Positive transfectants were selected on WR99210 (Walter Reed Army Institute until detectable by microscopy, at which point parasites were cycled on and off WR99210 for 6 x 21-day periods. Transfectants were subsequently cloned by limiting dilution and validated by Southern blotting.

### Parasite growth assays

To test PfRh4 function in the transgenic W2mef parasite lines, they were grown in neuraminidase (nm) (66.7 mU/mL, Calbiochem)-treated or non-treated erythrocytes at 2% haematocrit in RPMI complete media in 96-well microtitre plates (Becton Dickinson). Parasites were added to achieve 0.2% initial parasitaemia, and allowed to expand for 96 hours. They were stained with ethidium bromide to stain nuclear material (Bio-rad) for quantitation of erythrocytes infected with schizont stages by FACS (FACSCalibur, Becton Dickinson). At least 50,000 cells were counted from each well, and the erythrocyte population gated to determine parasitaemia using only late-trophozoite to schizont-infected erythrocytes (Flowjo software, Tree Star). Paired one-tailed t-tests with a threshold held at p = 0.05 were performed to assess the significance of growth differences. For each line, growth in neuraminidase-treated erythrocytes was calculated relative to growth in untreated erythrocytes (control).

### Parasite progression assays

3D7-PfCK2DD parasites were synchronized using sorbitol treatment. For Fig 3D, parasite cultures were grown at 3% parasitemia in 4% haematocrit. Shield-1 was removed when parasites were 0–4 hour rings (t = 0). Parasite cultures were sampled every two hours from 16 to 26 hours and 40 to 50 hours to follow their progression through the cell cycle. Briefly, 200 *μ*l of infected erythrocytes were fixed in 100 *μ*l of 0.25% glutaraldehyde for 30 mins. The samples were spun at 1200 rpm for 2 mins and the pellet was permeabilized with 100 *μ*l of 0.25% Triton X-100 for 5 mins followed by treatment with 100 *μ*l of 0.5 mg/ml RNase A (Invitrogen) for 30 mins. After washing, the samples were resuspended in 100 *μ*l of 5x SYBR Green and incubated in the dark for 10 mins. All samples were resuspended in 1 ml PBS for flow cytometry (FACSCalibur, Becton Dickinson). 50,000 cells were counted and analysed using Flowjo software, Tree Star. For Fig 3E, Shield-1 was removed when parasites were 24 to 26 hour and sampled every hour from 42 to 48 hours. Parasites were smeared onto slides and stained with Giemsa. Populations of rings, fully segmented schizonts and late schizont were counted by visualizing the parasites using light microscopy.

### Live cell imaging

Highly synchronous parasite cultures (5% parasitemia and 4% hematocrit) were diluted 1/25 in media from which 2 ml was placed into a 35 mm Fluorodish (World Precision Instruments) and allowed to settle to produce a monolayer. For neuraminidase treatment custom made 50 μl dishes were used. Neuraminidase at 0.2U/mL (Sigma) was added directly to the parasitized erythrocytes for 30 mins at 37°C. All live cell experiments were performed at 37°C on a Zeiss AxioObserver Z1 fluorescence microscope equipped with humidified gas chamber (94% N_2_, 1% O_2_, and 5% CO_2_). Time-lapse videos were recorded with high-resolution AxioCam MRm camera at 4 frames per second. ImageJ and Prism (Graphpad) were used to perform image and statistical analyses.

### Scoring of deformation

Contact between the merozoite and erythrocyte was defined as interactions where the erythrocyte and merozoite maintained physical contact for two frames (0.25 seconds) or longer. A deformation score of: 0 = sustained contact but no deformation, 1 = weak deformation at the point of contact, 2 = strong deformation with the erythrocyte membrane extending up sides of the embedded merozoite, and effects of deformation no longer strictly local to the merozoite, and 3 = extreme deformation with the deeply embedded merozoite partially covered by the erythrocyte membrane and extremely strong deformation distant from the merozoite to the point of distortion of erythrocyte borders [28].

## Supporting Information

S1 Fig *In vitro* kinase assay using CK2, GSK3, PK7, Map2 and PK6 kinase in the presence of histone HI, casein or maltose binding protein (MBP) as substrate. MW represents the molecular weight ladder.
(TIF)

S2 Fig (A) Schematic of the PfCK2 targeting vector. Single-crossover homologous recombination resulted in the integration of the HA-tag and destabilising domain (DD) into genomic PfCK2α. (B) Southern blot of transgenic PfCK2-HA-DD parasites. Genomic DNA was cut with EcoRI and Kpn1 and probed with a PfCK-specific probe. The expected sizes are shown in A. (C) Time course of 3D7-PfCK2DD strain in the presence and absence of Shield-1. Shield-1 was removed at the ring stage of a synchronous parasite population, Day 0 (5% parasitemia). PfCK2 expression was detected using anti-HA antibody. (D) Densitometry analysis was performed on the western blot depicting the regulation of PfCK2-HA-DD as shown in C (TIF)

S1 Movie Movie of Rh4-mut4 tail merozoites attempting to invade neuraminidase-treated erythrocytes. The merozoites attach and often weakly deform the neuraminidase-treated erythrocyte surface as they moved over it but rarely penetrate it.
(MOV)

S2 Movie Movie of Rh4-AMA1 tail merozoites attempting to invade neuraminidase-treated erythrocytes. The merozoites attach and often weakly deform the neuraminidase-treated erythrocyte surface as they moved over it but rarely penetrate it.
(MOV)

S3 Movie Movie of Rh4-175 tail merozoites attempting to invade neuraminidase-treated erythrocytes. The merozoites attach and often weakly deform the neuraminidase-treated erythrocyte surface as they moved over it but rarely penetrate it.
(MOV)

S4 Movie Movie of Rh4-WT tail merozoites attempting to invade neuraminidase-treated erythrocytes. They were unable to strongly deform neuraminidase-treated erythrocytes but were still able to invade which stands in contrast to previous data positively correlating successful invasion with strong deformation.
(MOV)
