# Supplementary figures and images for: Plasmodium falciparum Adhesins Play an Essential Role in Signalling and Activation of Invasion into Human Erythrocytes
Source: PLoS Pathog. 2015 Dec 22;11(12):e1005343. doi: 10.1371/journal.ppat.1005343 (PMC4687929; doi:10.1371/journal.ppat.1005343)

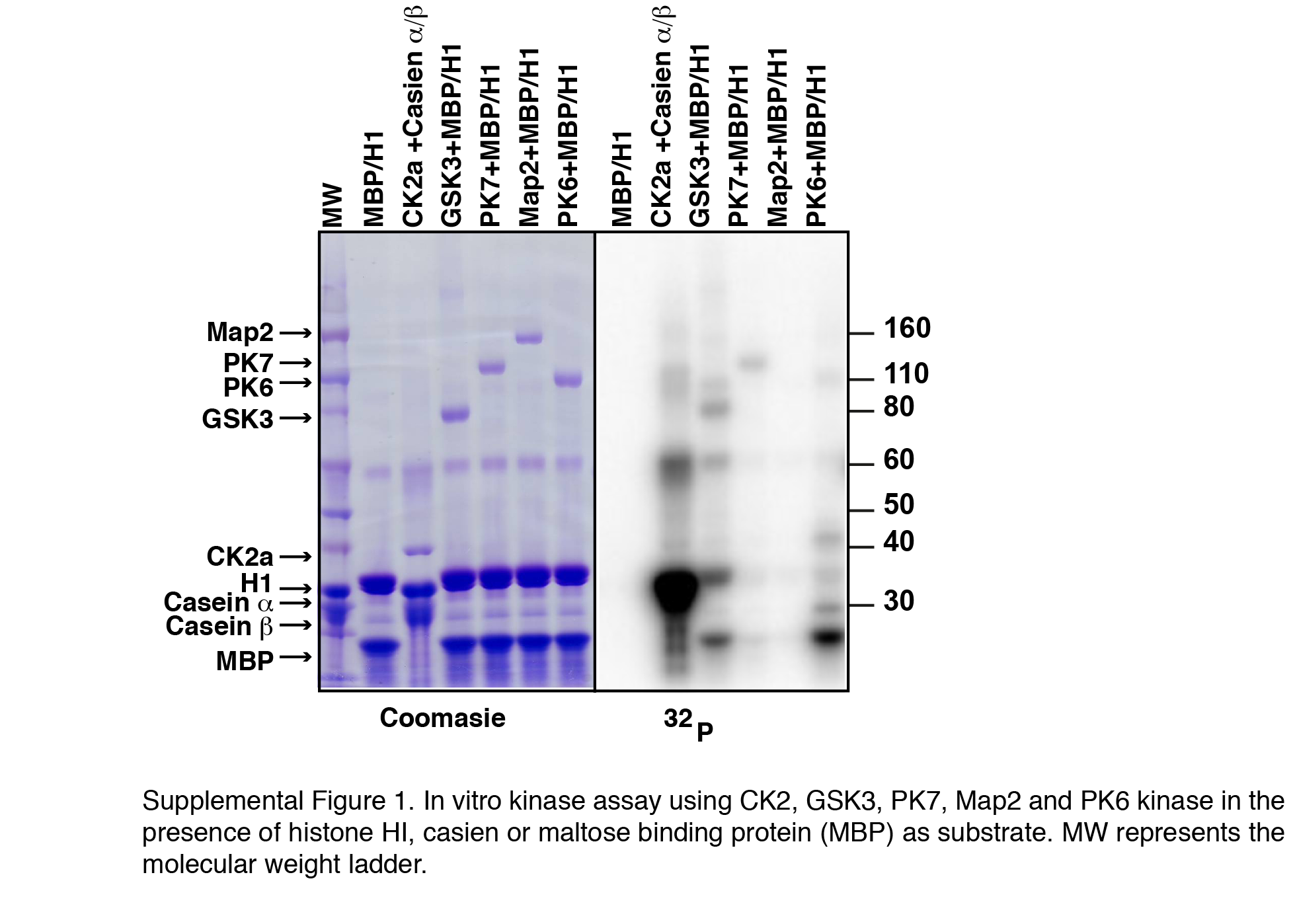

Supplement: S1 Fig — MW represents the molecular weight ladder. (TIF) [file ppat.1005343.s001.tif]

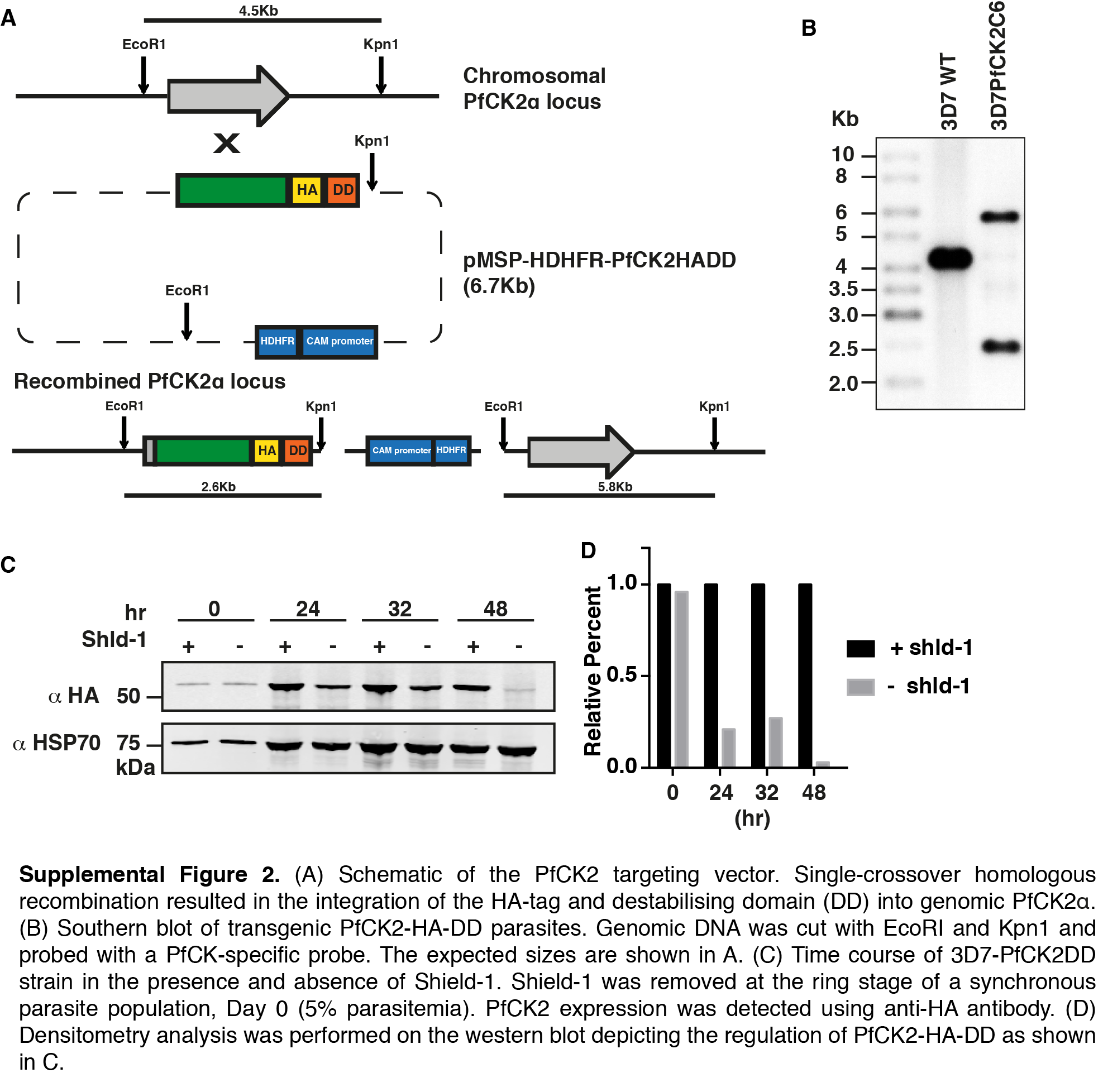

Supplement: S2 Fig — (TIF) [file ppat.1005343.s002.tif]
